# Branched-chain amino acids and L-alanine supplementation ameliorate calcium dyshomeostasis in sarcopenia: New insights for nutritional interventions

**DOI:** 10.3389/fphar.2024.1393746

**Published:** 2024-06-19

**Authors:** Elena Conte, Paola Mantuano, Brigida Boccanegra, Paola Imbrici, Giorgia Dinoi, Roberta Lenti, Ornella Cappellari, Donato Cappetta, Antonella De Angelis, Liberato Berrino, Heather Gordish-Dressman, Gianluca Bianchini, Andrea Aramini, Marcello Allegretti, Antonella Liantonio, Annamaria De Luca

**Affiliations:** ^1^ Section of Pharmacology, Department of Pharmacy-Drug Sciences, University of Bari “Aldo Moro”, Bari, Italy; ^2^ Department of Biological and Environmental Sciences and Technologies, University of Salento, Lecce, Italy; ^3^ Department of Experimental Medicine, University of Campania “Luigi Vanvitelli”, Naples, Italy; ^4^ Center for Genetic Medicine Research, Children’s National Medical Center, Washington, DC, United States; ^5^ Research & Early Development, Dompé farmaceutici S.p.A., L’Aquila, Italy

**Keywords:** branched-chain amino acids, sarcopenia, calcium homeostasis, skeletal muscle, L-alanine

## Abstract

**Introduction:** During aging, sarcopenia and decline in physiological processes lead to partial loss of muscle strength, atrophy, and increased fatigability. Muscle changes may be related to a reduced intake of essential amino acids playing a role in proteostasis. We have recently shown that branched-chain amino acid (BCAA) supplements improve atrophy and weakness in models of muscle disuse and aging. Considering the key roles that the alteration of Ca^2+^-related homeostasis and store-operated calcium entry (SOCE) play in several muscle dysfunctions, this study has been aimed at gaining insight into the potential ability of BCAA-based dietary formulations in aged mice on various players of Ca^2+^ dyshomeostasis.

**Methods:** Seventeen-month-old male C57BL/6J mice received a 12-week supplementation with BCAAs alone or boosted with two equivalents of L-alanine (2-Ala) or with dipeptide L-alanyl-L-alanine (Di-Ala) in drinking water. Outcomes were evaluated on *ex vivo* skeletal muscles indices vs. adult 3-month-old male C57BL/6J mice.

**Results:** Ca^2+^ imaging confirmed a decrease in SOCE and an increase of resting Ca^2+^ concentration in aged vs. adult mice without alteration in the canonical components of SOCE. Aged muscles vs*.* adult muscles were characterized by a decrease in the expression of ryanodine receptor 1 (RyR1), the Sarco-Endoplasmic Reticulum Calcium ATPase (SERCA) pump, and sarcalumenin together with an alteration of the expression of mitsugumin 29 and mitsugumin 53, two recently recognized players in the SOCE mechanism. BCAAs, particularly the formulation BCAAs+2-Ala, were able to ameliorate all these alterations.

**Discussion:** These results provide evidence that Ca^2+^ homeostasis dysfunction plays a role in the functional deficit observed in aged muscle and supports the interest of dietary BCAA supplementation in counteracting sarcopenia-related SOCE dysregulation.

## 1 Introduction

Aging is considered an important risk factor for many age-associated conditions, including sarcopenia (Khan et al., 2017), which is a progressive and generalized skeletal muscle condition that contributes to typical reduced mobility and loss of independence in the elderly (Batsis et al., 2014; Wiedmer et al., 2021). The age-related decline in skeletal muscle mass can be related to many possible causes, including anabolic dysfunction (Léger et al., 2008), satellite cell senescence (Chaib et al., 2002), and low-grade chronic inflammation (Schaap et al., 2009). Particularly, the alteration of muscle protein metabolism is responsible for the negative protein balance in relation to protein synthesis and degradation (Katsanos et al., 2005; Morley, 2016). Importantly, aging has myofiber phenotype-dependent effects, differently affecting fibers expressing the slow myosin heavy chain (MHC) I *versus* the fast MHC II (Grosicki et al., 2022), with the latter’s showing a profound age-related decrement in the size and contractile function. Considering the growing elderly population, the prevalence of sarcopenia will certainly increase in the coming years. In this context, current strategies are mainly focused on exercise, nutrition, and medications or the combination of the three remedies for promoting active aging (Chaib et al., 2002; Childs et al., 2017; Torcinaro et al., 2022).

The use of dietary supplements to support muscle protein synthesis is considered a feasible approach for counteracting muscle loss during aging (Lin et al., 2021). In particular, dietary supplementation of branched-chain amino acids (BCAAs: leucine, valine, and isoleucine) has been reported as beneficial (Argiles et al., 2015; Banfi et al., 2018; Boccanegra et al., 2020) in relation to the key role of these three essential amino acids (EAAs) in the regulation of protein synthesis (Bifari and Nisoli, 2017). Furthermore, BCAAs are known to play a role in insulin secretion, ATP synthesis (Brosnan and Brosnan, 2006), and the prevention of atrophy associated with cancer cachexia in mice (Eley et al., 2007). These amino acids are the only ones capable of initiating signal transduction mechanisms important for translation initiation (Yoshizawa, 2004). Among the three BCAAs, leucine is the most potent in stimulating muscle protein synthesis by activating the mammalian target of the rapamycin complex 1 (mTORC1) pathway and, in parallel, inhibiting protein degradation (Maki et al., 2012).

By using murine models, we have recently demonstrated that an oral supplementation with BCAAs (in a 2:1:1 ratio) ameliorated muscle function while increasing muscle mass in various conditions, such as physiological exercise, disuse-related atrophy, and age-related sarcopenia. The effects were especially evident with formulations boosted with L-alanine (L-ALA), the main amino acid derived from BCAA catabolism, particularly in the form of two equivalents (2ALA) or as a dipeptide (L-alanyl-L-alanine, Di-ALA) (Mantuano et al., 2020; Mantuano et al., 2021a; Mantuano et al., 2023). In this regard, the reduction of the histology markers of atrophy and the amelioration of *in vivo* and *ex vivo* muscle weakness observed in the aged mouse model further support the interest of supplement-based BCAAs to contrast sarcopenia (Mantuano et al., 2023). However, the molecular mechanisms underlying the ability of BCAAs to counteract functional age-related muscle alterations are unclear.

The regulation of cytosolic calcium levels in skeletal muscle fibers is crucial for proper contraction (Kuo and Ehrlich, 2015). In this context, a fine equilibrium is needed between excitation and dihydropyridine-coupled calcium release by sarcoplasmic reticulum *via* ryanodine receptor (RyR) channels, calcium reuptake by the Sarco-Endoplasmic Reticulum Calcium ATPase (SERCA) pump, and store-operated calcium entry (SOCE). SOCE is known to involve canonical components such as stromal interaction molecule 1 (STIM1) and calcium release-activated calcium channel protein (ORAI1), plus other proteins such as mitsugumin 53 (MG53) and mitsugumin 29 (MG29), which are emerging as novel key players in the SOCE mechanism (Gruszczynska-Biegala et al., 2021).

SOCE is important in limiting fatigue during repetitive high-frequency stimulation (Wei-Lapierre et al., 2013; Boncompagni et al., 2017; Cho et al., 2017; Michelucci et al., 2020; Protasi et al., 2023), whereas its alteration has been associated with the progression of some muscular disorders, including muscular dystrophy (Edwards et al., 2010; Harisseh et al., 2013) and cachexia (Conte et al., 2017), as well as aging. In particular, together with an increase in cytosolic Ca^2+^ concentration (Fodor et al., 2020; Mijares et al., 2021), a reduction in SOCE activity contributes to age-related muscle weakness (Zhao et al., 2008; Thornton et al., 2011).

In light of the above considerations, the main aim of this study was to investigate if BCAA formulations may have a role in modulating Ca^2+^ intracellular levels and the SOCE mechanism of sarcopenic skeletal muscle. We performed *ex vivo* cytofluorometry and biochemical investigations on skeletal muscles from aged male C57BL/6J mice treated with BCAAs alone or combined with boosting 2ALA or Di-ALA, as a direct follow-up of our previous work (Mantuano et al., 2023), focusing the attention on key molecular components involved in cellular calcium homeostasis.

## 2 Materials and methods

### 2.1 Ethical statement for animal studies

This study was approved by the National Ethics Committee for Research Animal Welfare of the Italian Ministry of Health (authorization no. 1119/2020-PR). All the experiments were conducted in conformity with the Italian Guidelines for Care and Use of Laboratory Animals (D.L.116/92) and the European Directive (2010/63/EU), as well as in compliance with the ARRIVE guidelines. Accordingly, the present experiments were ethically conducted in continuity with the functional and morphological assessments whose results were published in the study by Mantuano et al. (2023) using spared samples from the same set of animals.

### 2.2 Animal groups and treatment

**FIGURE 1:**
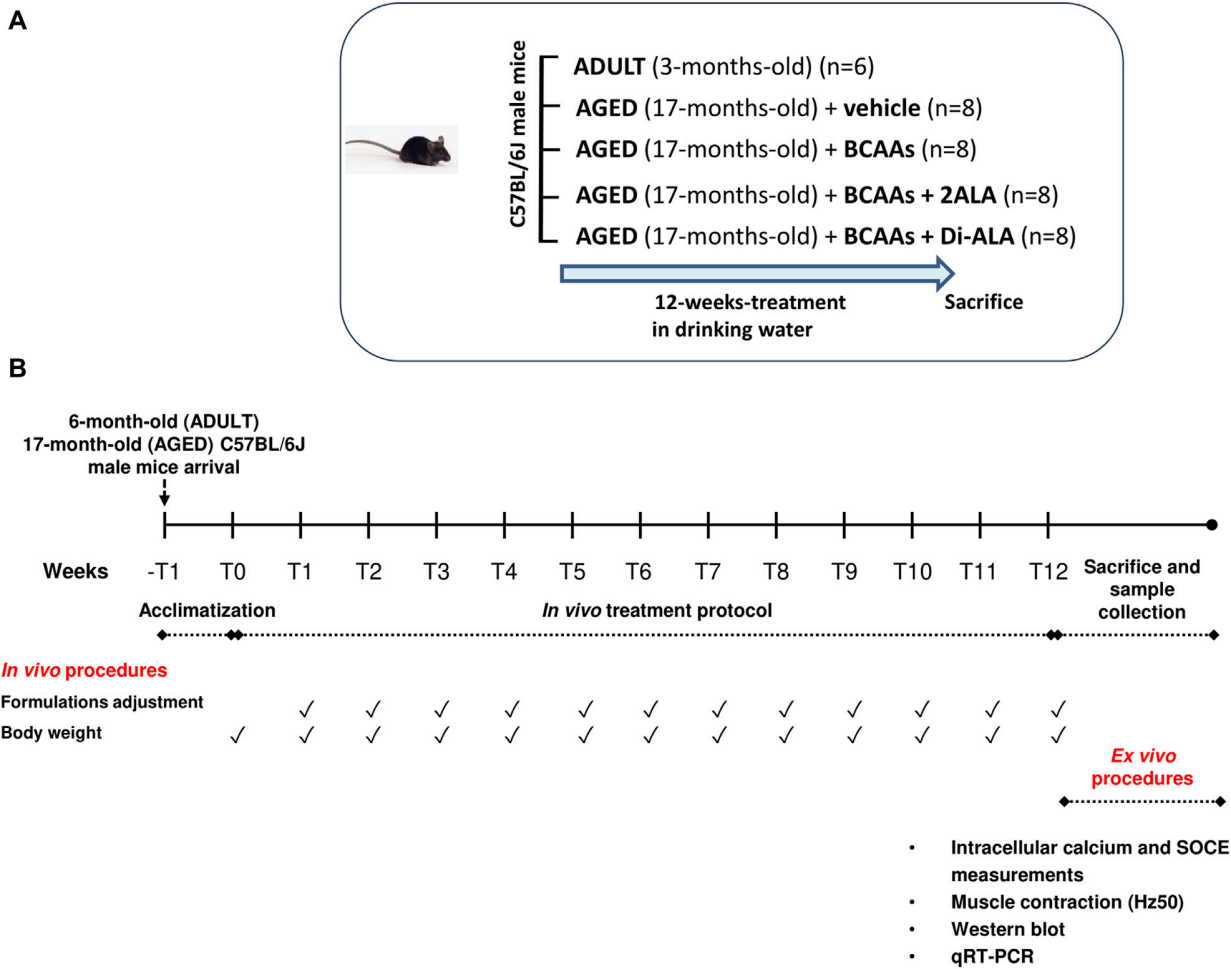
Schematic representation of the experimental protocol. **(A)** 17-month-old male C57BL/6J mice received 12-week treatment (20-month-old at the end of treatment) with vehicle (AGED + vehicle) branched-chain amino acids (BCAAs, AGED + BCAAs) alone or boosted with two equivalents of L-alanine (2ALA, AGED + BCAAs + 2ALA) or with dipeptide L-alanyl-L-alanine (Di-ALA, AGED + BCAAs + Di-ALA), in drinking water. Outcome was evaluated on *in vivo*/*ex vivo* indices vs. ADULT 3-month-old male C57BL/6J mice (6-month-old at the end of treatment). **(B)** Scheme illustrating experimental design and timeline of the study. After a week of acclimatization, mice received 12-weeks of treatment, as previously described, in drinking water. Each formulation was prepared once a week. Water intake and body weight were monitored once a week to allow the adjustment of administered doses. Once the 12-week treatment had ended, mice were sacrificed for *ex vivo* procedures.

Seventeen-month-old (n = 32) and adult 3-month-old (n = 6) male C57BL/6J mice (Charles River Laboratories, Calco Italy), for a total of n = 38 animals, were used. All mice were first acclimatized and successively divided into experimental groups, as described in the study by Mantuano et al. (2023). Briefly, after acclimatization, cohorts of aged mice (4 groups, each consisting of n = 8 animals) were randomized to receive a 12-week treatment with a vehicle (filtered tap water), the BCAA formulation, or one of the modified formulations—BCAAs + 2ALA or BCAAs + Di-ALA—in drinking water, whereas a group of adult 3-month-old male C57BL/6J mice (n = 6) was used as the control to better evaluate the alteration of specific functional parameters not yet assessed in the aged mouse model (Figure 1). Each formulation was prepared once a week by dissolving the powder of the amino acid mixture in filtered tap water to obtain the desired final dose: for the BCAA mixture (weight ratio of L-Leu:L-Ile:L-Val 2:1:1), we obtained a final dose of 656 mg/kg, for BCAAs + 2ALA (L-Leu:L-Ile:L-Val:L-ALA 2:1:1:2), we obtained a final dose of 984 mg/kg, and for BCAAs + DiALA (L-Leu:L-Ile:L-Val:Di-ALA 2:1:1:2), we obtained a final dose of 984 mg/kg (Mantuano et al., 2023). Water intake was monitored once a week to allow the adjustment of administered doses considering the amount of water consumed per cage, divided for the number of mice in the cage, and normalized to their mean body weight. Once the 12-week treatment had ended, mice were deeply anesthetized by intraperitoneal (ip) injection with a cocktail of ketamine (100 mg/kg) and xylazine (16 mg/kg) to allow skeletal muscle dissection (Figure 1). Specifically, our study focused on fast-twitch muscles, i.e., the muscles more affected by the aging process as they mainly contain fibers expressing the MHC II isoform (Deschenes et al., 2013; Grosicki et al., 2022). The use of multiple techniques to determine the parameters under investigation (calcium homeostasis, gene and protein expression, and muscle contractile properties) forced us to use different fast-twitch muscles, such as extensor digitorum longus (EDL), flexor digitorum brevis (FDB), and gastrocnemius (GC) muscles. These muscles were used immediately for cytofluorimetric studies or frozen in liquid nitrogen and stored at –80 °C for further gene and protein expression analysis.

### 2.3 *Ex vivo* isometric contraction recordings

Extensor digitorum longus (EDL) and soleus (SOL) muscles from the left hind limb were isolated and adequately prepared to be used for contractile recordings, as described in full detail by Mantuano et al. (2023). Briefly, each muscle was placed into a horizontal muscle bath (mod. 809B-25, Aurora Scientific Inc.–ASI–Aurora, ON, Canada) containing 25 mL of isotonic Ringer’s solution (mM: NaCl 148, KCl 4.5, CaCl_2_ 2.0/2.5, MgCl_2_ 1.0, NaH_2_PO_4_ 0.44, NaHCO_3_ 12.0, and glucose 5.55; pH 7.2–7.4; 27 ± 1°C), continuously gassed with a mixture of 95% O_2_ and 5% CO_2_, and thermostatically maintained at 27°C ± 1°C. One tendon was fixed at the bottom of the chamber, and the other tendon was fixed to a force transducer (mod. 300C-LR, Aurora Scientific Inc.–ASI–Aurora, ON, Canada). Electrical field stimulation was obtained by two axial platinum electrodes closely flanking the muscle, connected to a high-power bi-phase stimulator (mod. 701C, Aurora Scientific Inc.–ASI–Aurora, ON, Canada). Data were acquired *via* a dedicated signal interface and software (mod. 604A plus DMCv5.415, Aurora Scientific Inc.–ASI–Aurora, ON, Canada). After equilibration (∼30 min), muscle preparations were stretched to their optimal length (L0, measured with an external caliper). Then, the single twitch tension and tetanic contractions at increasing frequencies (10–250 Hz) were elicited according to validated protocols of stimulation (Mantuano et al., 2020; Mantuano et al., 2021b; Mantuano et al., 2023). This allowed us to calculate using ASI software DMAv5.201 and SigmaPlot 10.0, the Hz50, a calcium-sensitive parameter indicating the frequency at which 50% of maximal sP0 is produced.

### 2.4 Fiber isolation and Fura2 fluorescence measurements

Cytofluorimetric experiments were performed in adult and aged muscle fibers from flexor digitorum brevis (FDB) dissociated using collagenase in normal physiological Ringer’s solution, according to previous studies (De Backer et al., 2002; Conte et al., 2021). Briefly, FDB muscles were removed from mice and transferred to a dish containing oxygenated Ringer’s solution (mM: NaCl 148; KCl 5.5; MgCl_2_ 1; CaCl_2_ 2; NaHPO_4_ 0.44; NaHCO_3_ 12; glucose 5.55) to remove residual connective tissue plantaris tendons and then into a dissecting dish containing oxygenated Ringer’s solution and 0.2% type IV collagenase (Sigma-Aldrich, United States, C-5138) for 45 min at 37°C. The pH of all solutions was adjusted to 7.3–7.4 by bubbling them with 95% O_2_/5% CO_2_. After incubation, fibers were washed with Ringer’s solution and gently dissociated by several passages through a series of Pasteur pipettes of progressively decreasing diameter. Successively, dissociated fibers were sampled and transferred into a rectangular PRESTIGE 24 × 50 mm glass coverslip (Syntesys disposable labware, cod.372114), coated with PathClear™ extracellular matrix basement membranes (Cultrex, BioTechne, Minneapolis, MN, United States, cod. #3432–005-01). The coverslips were kept in an incubator with 5% CO_2_ at 30 °C, and the adhesion of the fibers to the matrix occurred within 2 h. At the end, the coverslips with adhered dissected fibers were transferred into a modified RC-27NE experimental chamber (Warner Instrument Inc., Hamden, United States) for Fura-2 loading and intracellular calcium analysis.

Calcium measurements were performed using the membrane-permeable Ca^2+^ indicator Fura-2 acetoxymethyl ester (Fura-2AM, Thermo Fisher Scientific, Waltham, United States, cod.15455039). Loading of muscle fibers was performed for 1 h in an incubator, with 5% CO_2_ at 30°C in normal physiological Ringer’s solution containing 5 μm Fura-2AM mixed with 0.05% (v/v) Pluronic F-127 (Thermo Fisher Scientific, Waltham, United States, cod. P3000MP). After loading, muscle fibers were washed, and the experimental chamber was placed on the stage of an inverted Eclipse TE300 microscope (Nikon, Japan) with a 40X Plan Fluor objective (Nikon, Japan) for cytofluorimetric analyses. The QuantiCell 900 integrated imaging system (VisiTech International Ltd., Sunderland, United Kingdom) was used for fluorescence measurements, as previously described (Fraysse et al., 2003; Liantonio et al., 2014).

Pairs of background-subtracted images of Fura-2 fluorescence (510 nm) excited at 340 and 380 nm were acquired at rest, and ratiometric images (340/380 nm) were calculated for each dissected muscle fiber using QuantiCell 2000 software version 2.0e (VisiTech International Ltd., Sunderland, United Kingdom) and converted to calcium concentration (nM), after the calibration procedure, using the equation: [Ca^2+^]i = (R-Rmin)/(Rmax-R)*KD*β, where R is the ratio of the fluorescence excited at 340 nm to that excited at 380 nm; KD is the affinity constant of Fura-2 for calcium, which was taken as 145 nM; and β, Rmin, and Rmax were determined experimentally *in situ* in ionomycin-permeabilized muscle fibers, as previously described (Conte et al., 2017). Because calibration parameters of Fura-2 can depend on muscle type and experimental condition (Fraysse et al., 2003), we determined the parameters of Grynkiewicz’s equation in each muscle examined for accurate calculation of calcium concentration and used a unique batch of Fura-2 to guarantee no variation in KD between experimental conditions.

### 2.5 Store-operated calcium entry measurements

A part of dissected FDB fibers adhered on coverslips was used for the SOCE measurement, performed according to previous studies (Conte et al., 2021; Sakai-Takemura et al., 2023). Particularly, fibers were incubated with a calcium free-solution (mM: 148 NaCl, 4.5 KCl, 10 EGTA, 1 MgCl_2_, 0.44 NaH_2_PO_4_, 12 NaHCO_3_, and 5.5 glucose) and 2 μM thapsigargin (Sigma-Aldrich, United States, cod. T9033) to passively deplete Ca^2+^ stores. Successively, calcium-free solution was replaced with normal physiological solution containing calcium (mM: 148 NaCl, 4.5 KCl, 2.5 CaCl_2_, 1 MgCl_2_, 0.44 NaH_2_PO_4_, 12 NaHCO_3_, and 5.5 glucose) to allow extracellular calcium entry in the cell following the concentration gradient (SOCE protocol). The pH of all solutions was adjusted to 7.3–7.4 by bubbling them with 95% O_2_/5% CO_2_. All chemicals cited above were purchased from Sigma (St. Louis, MO, United States).

### 2.6 Total RNA purification and real-time PCR

Gene expression analysis was performed in gastrocnemius (GC) muscles of different experimental groups according to a previous study (Boccanegra et al., 2023a). For each muscle sample, total RNA was isolated with TRIzol (Thermo Fisher Scientific, Waltham, United States, cod.10296028). RNA quantification and quality validation (260/280 and 260/230 ratios) were evaluated by using a spectrophotometer (ND-1000 NanoDrop, Thermo Fisher Scientific). Reverse transcription was performed using an iScriptTM gDNA CLR cDNA kit (Bio-Rad, Hercules, CA, United States, cod.1725034). Real-time PCR was performed using the Bio-Rad CFX Connect instrument and custom pre-loaded PCR plates. In addition to the selected genes, all pre-loaded plates included the following controls: DNA contamination control (Bio-Rad, Hercules, CA, United States, Unique assay ID: qMmuCtlD0001004), a positive PCR control (Bio-Rad, Hercules, CA, United States, Unique assay ID: qMmuCtlD0001003), and a reverse transcription control assay (Bio-Rad, Hercules, CA, United States, Unique assay ID: qMmuCtlD0001001). Two technical replicates per biological replicates were performed for each experiment. Amplification of cDNA (3 ng) was performed in a total volume of 20 μL of SsoAdvanced Universal SYBR Green Suprmix (Bio-Rad, Hercules, CA, United States, cod #1725271). All qPCR analyses performed using SYBR Green were conducted at 50°C for 2 min and 95°C for 2 min, and then 40 cycles of 95°C for 15 s and 60°C for 1 min.

qPCR primer assays (Bio-Rad, Hercules, CA, United States) were ordered with the following unique assay IDs: *Orai1:* qMmuCID0020628; *Cacna1s:* qMmuCID0021135; *Casq1:* qMmuCID0010641; *Ryr1:* qMmuCID0023154; *Srl:* qMmuCID0017758; *Atp2a1:* qMmuCID0027023; *Atp2a2:* qMmuCID0005528; *Stim1*: qMmuCID0021406; *Sypl2*: qMmuCID0010947; *Trpc1:* qMmuCID0013199; and *Trim72:* qMmuCID0007333. Relative mRNA concentration of the target genes was normalized to the corresponding β-actin internal control, which was the more stable housekeeping among *β-actin* (unique assay ID: qMmuCED0027505), glyceraldehyde-3-phosphate dehydrogenase (GAPDH, unique assay ID: qMmuCED0027497), and hypoxanthine phosphoribosyltransferase 1 (*Hprt1*, unique assay ID: qMmuCID0005679), and quantified by the 2^−ΔΔct^ method (Livak and Schmittgen, 2001). All primers were validated by Bio-Rad laboratories by analyzing the amplification plot, melt peak, and standard curve. At the end of all PCR cycles, a melt curve analysis was performed to confirm the specificity of primer annealing.

### 2.7 Western blot analysis

Total proteins were extracted from gastrocnemius (GC) muscles of different experimental groups, as described by Boccanegra et al. (2023b) with some modifications. Briefly, all muscles were homogenized in ice-cold buffer containing 10 mM Tris·HCl (pH 7.4 at 4°C), 1% Triton X100, 10% glycerol, 150 mM NaCl, 5 mM EDTA, 1 mM sodium vanadate, and protease inhibitor cocktail (Roche, Cat# 5056489001). Homogenates were centrifuged at 1,500 *g* for 15 min at 4°C, and the supernatant was quantified using the BCA Protein Assay Kit (Sigma-Aldrich, United States, bicinchoninic acid solution, cod.1003278214; copper (III) sulfate solution, cod.1003274018) according to the manufacturer’s instructions. For immunoblot analysis, 40 μg of protein were separated on 10% Mini–PROTEAN TGX Stain-Free Protein Gels (Bio-Rad, Hercules, CA, United States, Cat# 4568033) and transferred onto stain-free polyvinylidene difluoride (PVDF) membranes (Bio-Rad, Hercules, CA, United States, Cat# 1704156) for 7 min at 1,3A–25 V (Trans-Blot ^®^ Turbo™ Transfer System, Bio-Rad, Hercules, CA, United States). The incubation in primary antibodies was carried out overnight at 4°C, and anti-mouse (1:5000 anti-mouse IgG, Sigma-Aldrich, United States, Cat# A9044, RRID:AB_258431) for transient receptor potential channel 3 (TRPC3) and anti-rabbit (1:5000 anti-rabbit IgG, Bio-Rad, Hercules, CA, United States, Cat# 170–6515 (also 1706515), RRID:AB_11125142) for transient receptor potential channel 4 (TRPC4), mitsugumin 29 (MG29), and mitsugumin 53 (MG53) horseradish peroxidase-conjugated secondary antibodies were carried out at room temperature. Dilutions of primary antibodies were used as follows: TRPC3 (dilution 1:500, Santa Cruz Biotechnology Cat# sc-514670, RRID:AB_3086704), TRPC4 (dilution 1:700, Sigma-Aldrich Cat# SAB2108245, RRID:AB_3086705), MG29 (dilution 1:1,000, Sigma-Aldrich Cat #SAB3500069, RRID:AB_10604155), and MG53 (dilution 1:700, MyBioSource Cat# MBS8502881, RRID:AB_3086706).

Protein detection was conducted as described previously (Conte et al., 2020; Mantuano et al., 2021a). Briefly, the bands were visualized with a chemiluminescent substrate (Clarity Western ECL Substrate, Bio-Rad, Hercules, CA, United States, cat# 1705061), and signals were recorded with the ChemiDoc MP imaging system (Bio-Rad, Hercules, CA, United States). The evaluation of the relative amount of each analyzed protein was performed by relating the densitometric value of optical density (OD) units of each protein band to the OD units of the respective vinculin band (dilution 1:800, Thermo Fisher Scientific Cat #MA5-11690, RRID:AB_10976821) using Image Laboratory software version 6.1 (Bio-Rad Laboratories, Inc. United States). The software detects the chemiluminescence of each protein band obtaining the absolute signal intensity. The density volume was automatically adjusted by subtracting the local background.

### 2.8 Statistical analysis

All experimental data were expressed as the mean ± standard error of mean (SEM). All data followed a normal distribution by the Shapiro–Wilk test, being included in the 95% confidence interval of the mean, which allowed to apply the following parametric statistical analyses (Mantuano et al., 2020; Mantuano et al., 2021b; Mantuano et al., 2023). Single comparison between means by the unpaired Student’s t-test was only used to address differences between AGED + vehicle mice *versus* ADULT mice (*) as a stand-alone analysis in order to assess the presence of an aged phenotype on the parameters of interest. Multiple statistical comparisons among experimental groups (AGED + vehicle, AGED + BCAAs, AGED + BCAAs + 2ALA, and AGED + BCAAs + Di-ALA) were performed by one-way analysis of variance (ANOVA), with Dunnett’s test *post hoc* correction (°) using the GraphPad Prism version 8 program. *p* < 0.05 was statistically significant. The exclusion of specific samples from data analyses was only due to overt technical issues during experiments.

Whenever appropriate, the recovery score, which is an objective index directly indicating how much of the deficit is recovered (%) by a treatment, was calculated according to TREAT-NMD SOPs (ID Number M.1.1_001), as follows:

$$Recovery\;score=\frac{\text{treated}\;\text{AGED}\;\text{mice}-\text{untreated}\;\text{AGED}\;\text{mice}}{\text{ADULT}\;\text{mice}-\text{untreated}\;\text{AGED}\;\text{mice}}*100.$$

## 3 Results

### 3.1 Resting intracellular calcium and SOCE

**FIGURE 2:**
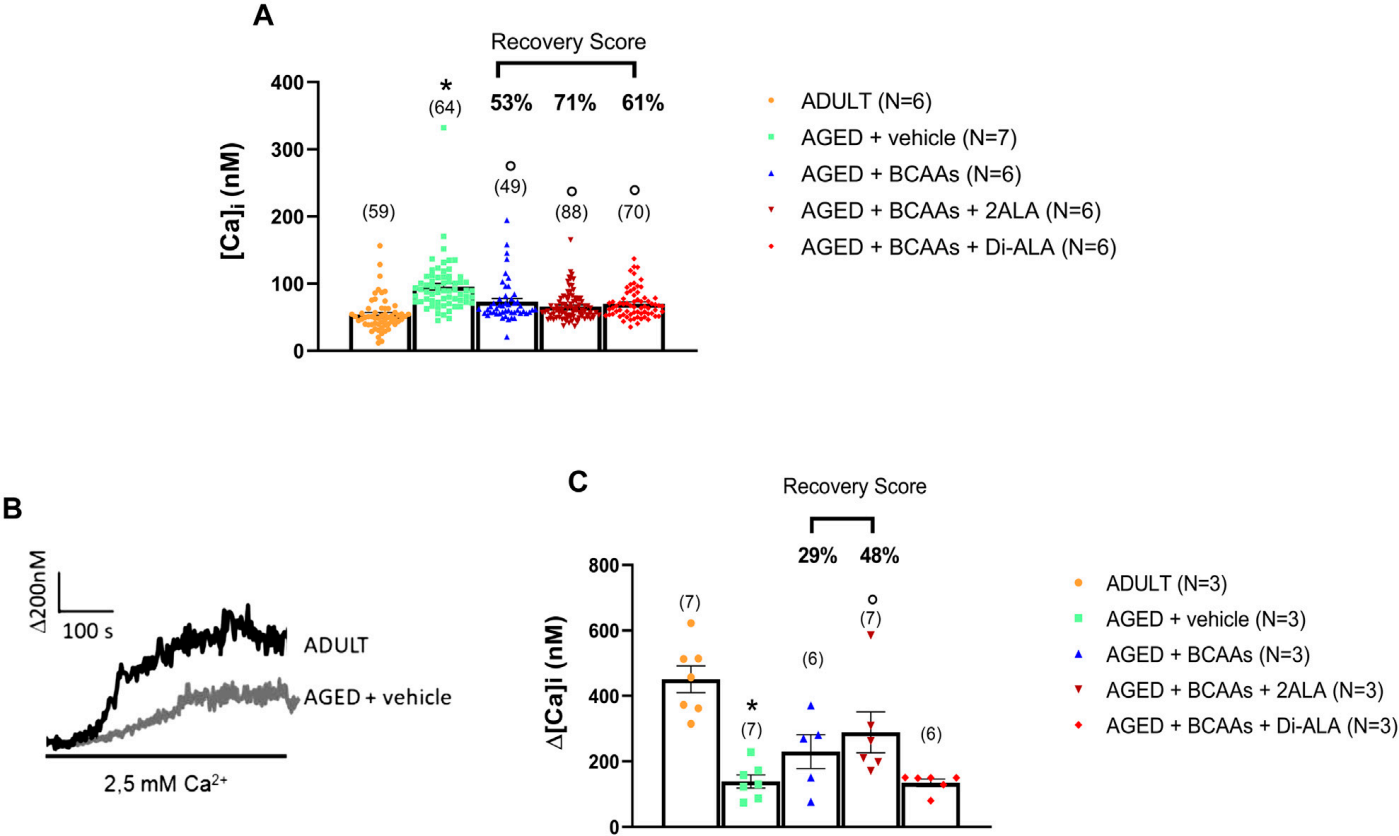
Calcium homeostasis characterization of adult, aged, and treated FDB fibers. **(A)** Resting calcium concentration measured in ADULT, AGED + vehicle, AGED + branched-chain amino acids (AGED + BCAAs), AGED + branched-chain amino acids +2 L-alanine (AGED + BCAAs + 2ALA), and AGED + branched-chain amino acids + L-alanyl-L-alanine (AGED + BCAAs + Di-ALA). A statistically significant difference was found by unpaired Student’s t-test for AGED + vehicle vs. ADULT (*p < 0.0001). A statistically significant difference was found among AGED mice groups by one-way ANOVA (F = 14.87; *p* < 0.0001). Dunnett’s *post hoc* test, used to compare each mixture-treated group to the vehicle group, is as follows: °vs. AGED + vehicle (*p* < 0.0001). Values are expressed as the mean ± SEM of the number of fibers analyzed (indicated in brackets over the bars) derived from a number of 6/7 mice in each group. **(B)** Representative traces of increased Ca^2+^ entry in store-depleted thapsigargin-treated muscle fibers after the addition of extracellular calcium (see SOCE protocol described in Materials and methods) in ADULT and AGED + vehicle. **(C)** Amplitude values of [Ca^2+^]i observed with the SOCE protocol in ADULT, AGED + vehicle, AGED + BCAAs, AGED + BCAAs + 2ALA, and AGED + BCAAs + Di-ALA. A statistically significant difference was found by unpaired Student’s t-test for AGED + vehicle vs. ADULT (*p < 0.0001). A statistically significant difference was found among AGED mice groups by one-way ANOVA (F = 3.589; *p* = 0,0318). Dunnett’s *post hoc* test, used to compare each mixture-treated group to the vehicle group, is as follows: °vs. AGED + vehicle (*p* < 0.035). Values are expressed as the mean ± SEM of the number of fibers analyzed (indicated in brackets over the bars) derived from a number of 3 mice in each group. The recovery score toward the ADULT value, calculated for each treated group, is indicated above the bars.

As expected (Fraysse et al., 2006; Mijares et al., 2021), the resting intracellular calcium concentration, [Ca^2+^]_i_, was significantly higher in FDB aged muscle fibers than in fibers from adult mice (AGED + vehicle: 94 ± 5 nM vs. ADULT: 53 ± 3 nM). Treatments with all mixtures were able to significantly prevent this increase by bringing the intracellular calcium levels to values almost overlapping those of the adult mice (recovery scores toward ADULT value: AGED + BCAAs 53%; AGED + BCAAs + 2ALA 71%; and AGED + BCAAs + Di-ALA 61% (Figure 2A).

To evaluate SOCE activity, SR Ca^2+^ stores of FDB muscle fibers were depleted with thapsigargin in the absence of extracellular Ca^2+^ and then extracellular Ca^2+^ was applied to muscle fibers to measure SOCE. As shown in Figures 2B, C, according to previous studies (Zhao et al., 2008; Thornton et al., 2011), aged muscle fibers showed an SOCE value significantly reduced with respect to adult muscle fibers (AGED + vehicle: 138 ± 19 nM vs. ADULT: 450 ± 50 nM). A trend of increased SOCE was observed with the treatment with BCAAs and BCAAs + 2ALA, with the latter formulation being the most effective (recovery scores toward ADULT value: AGED + BCAAs 29% and AGED + 2ALA 48%). In contrast, no significant effect was exerted by BCAAs + Di-ALA, with the SOCE value completely overlapping that of aged untreated mice.

**FIGURE 3:**
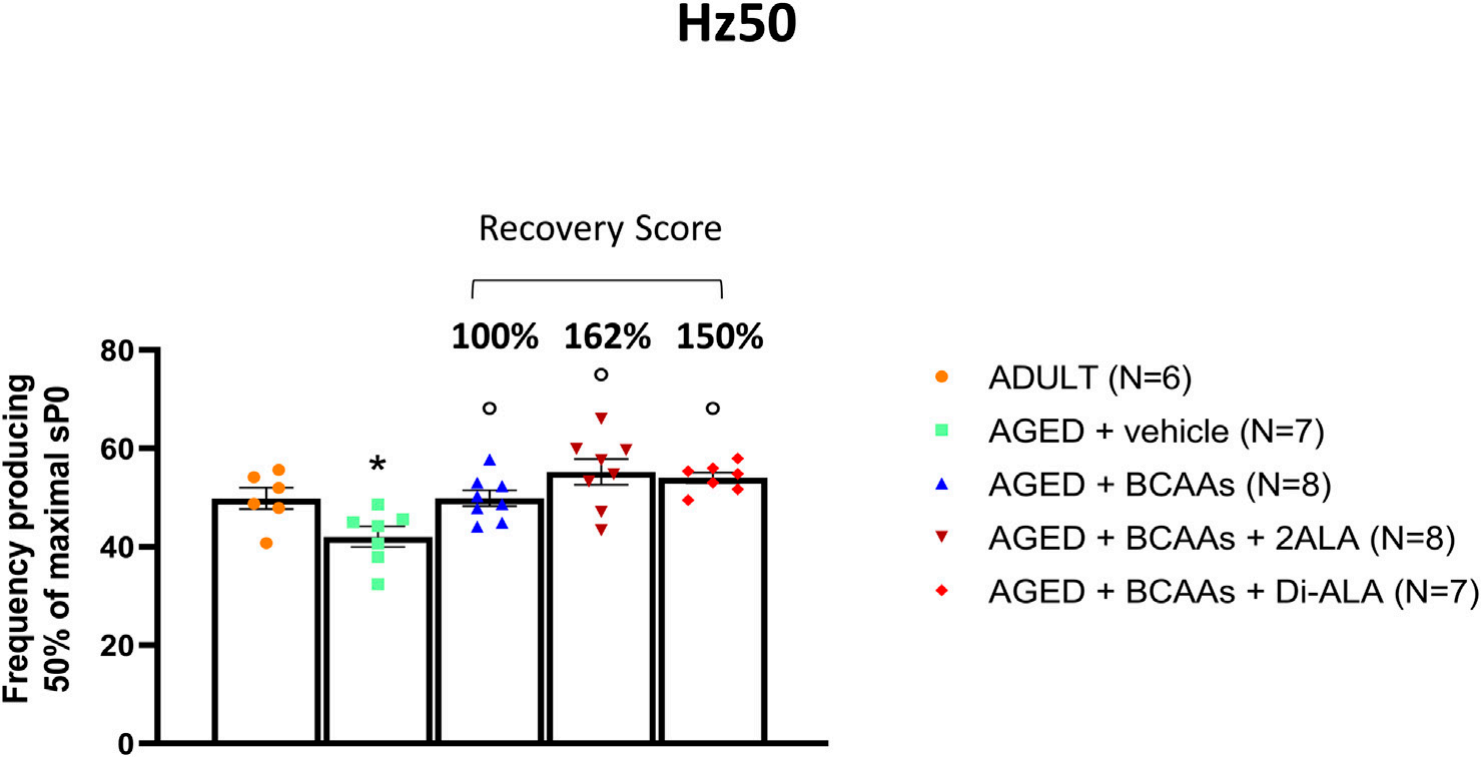
Hz50 of isolated EDL muscle. Values for Hz50, a contractile index related to calcium homeostasis indicating the frequency at which 50% of maximal isometric specific tetanic force (sP0) is produced, measured in extensor digitorum longus (EDL) muscles isolated from ADULT, AGED + vehicle, AGED + branched-chain amino acids (AGED + BCAAs), AGED + branched-chain amino acids +2 L-alanine (AGED + BCAAs + 2ALA), and AGED + branched-chain amino acids + L-alanyl-L-alanine (AGED + BCAAs + Di-ALA). All values are expressed as mean ± SEM from a number of 6/8 mice in each group. A statistically significant difference was found by unpaired Student’s t-test for AGED + vehicle vs. ADULT mice (*p* < 0.03). A statistically significant difference among AGED mice groups was found by one-way ANOVA (F = 8.9, *p* = 0.0003). Dunnett’s *post hoc* test, used to compare each mixture-treated group to the vehicle group, is as follows: °vs. AGED + vehicle (0.0002 < *p* < 0.03). The recovery score toward the ADULT value, calculated for each treated group, is indicated above the bars.

To investigate the outcome of the improved Ca^2+^ homeostasis at the functional level, we focused on calcium-dependent functional indices calculated during isometric contraction recordings in isolated fast-twitch muscle (i.e., EDL) and particularly on the frequency of stimulation to obtain a half maximal tetanic contraction (Hz50). In fact, in parallel with a significant decline in the maximal specific tetanic force (Mantuano et al., 2023), EDL muscles from aged mice also exhibited a significant shift of the Hz50 toward lower frequencies (i.e., a train of pulses of lower frequency is needed to get a half maximal contraction) with respect to adult mice (Figure 3). Importantly, a significant amelioration of Hz50 was observed in aged mice treated with all three formulations (Figure 3), with BCAAs + 2ALA being especially effective (recovery scores toward ADULT value: AGED + BCAAs 100%; AGED + BCAAs + 2ALA 162%; and AGED + BCAAs + Di-ALA 150%).

In parallel, Hz50 was not significantly altered in slow-twitch SOL muscles of aged mice compared to adult mice (26.8 ± 2.2 vs. 29.9 ± 4.9, respectively), further supporting the rationale to focus our analyses on fast-twitch muscle phenotype. Furthermore, no changes in calcium-sensitive kinetic indices were observed in either EDL or SOL muscle (data not shown).

### 3.2 Expression profile of genes involved in SOCE mechanism and calcium buffering

**FIGURE 4:**
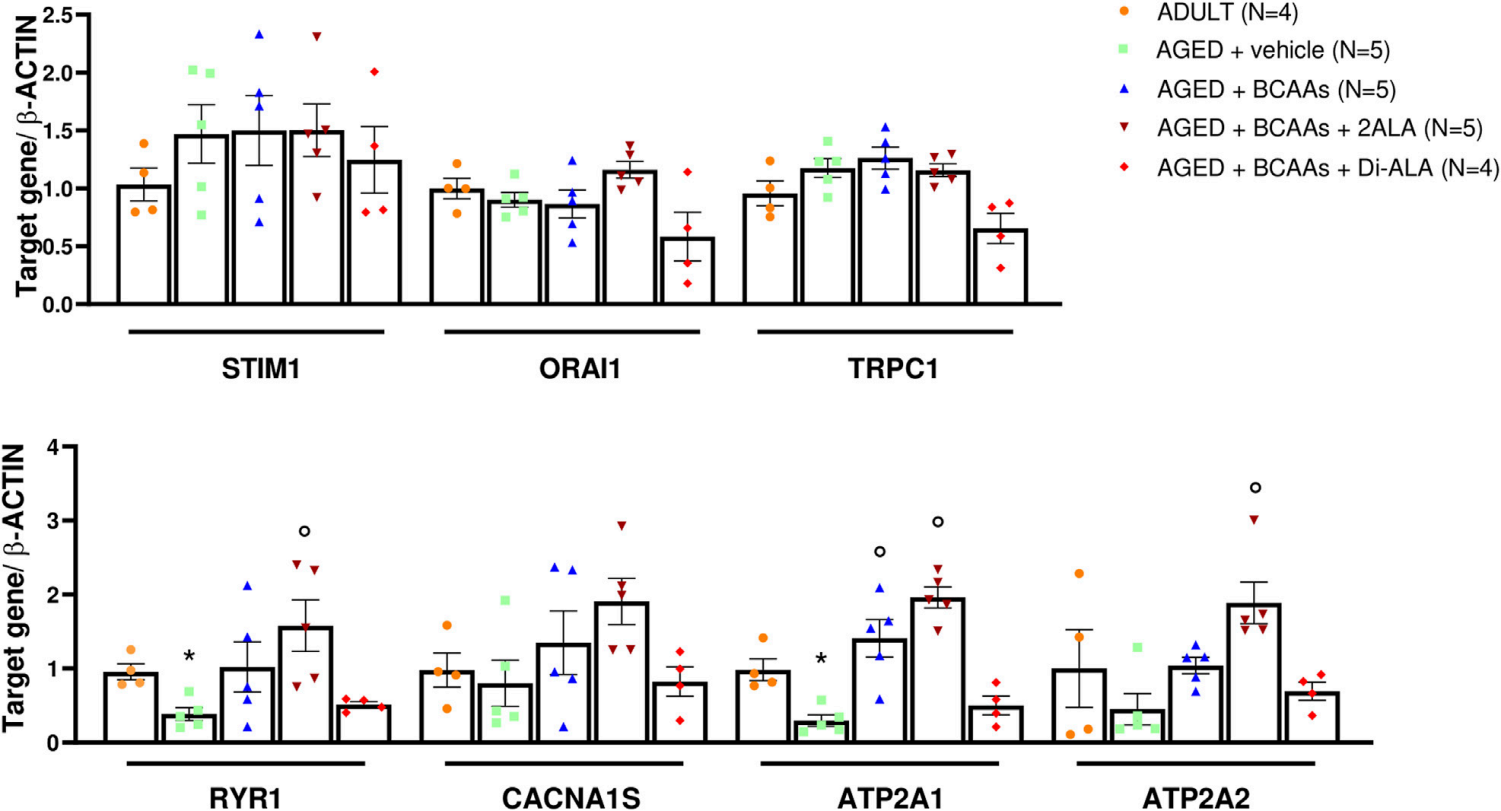
mRNA expression levels of selected genes involved in the SOCE mechanism. Relative content of mRNA levels for ORAI calcium release-activated calcium modulator 1 (*orai1*), stromal interaction molecule 1 (*stim1*), transient receptor potential cation channel subfamily C member 1 (*trpc1*) genes (upper panel), ryanodine receptor 1 (*ryr1*), calcium voltage-gated channel subunit alpha1 S (*cacna1s*) (encoding for dihydropyridine receptor, DHPR), ATPase sarcoplasmic/endoplasmic reticulum Ca^2+^ transporting 1 (*atp2a1*) (encoding for sarcoplasmic/endoplasmic reticulum calcium ATPase1, SERCA1), and ATPase sarcoplasmic/endoplasmic reticulum Ca^2+^ transporting 2 (*atp2a2*) (encoding for sarcoplasmic/endoplasmic reticulum calcium ATPase2, SERCA2) genes (bottom panel) involved in store-operated calcium entry (SOCE) mechanism and calcium homeostasis, respectively, normalized to *β-actin* gene in gastrocnemius (GC) muscles isolated from ADULT, AGED + vehicle, AGED + branched-chain amino acids (AGED + BCAAs), AGED + branched-chain amino acids +2 L-alanine (AGED + BCAAs + 2ALA), AGED + branched-chain amino acids + L-alanyl-L-alanine (AGED + BCAAs + Di-ALA) groups. A statistically significant difference was found by unpaired Student’s t-test for AGED + vehicle vs. ADULT (*p = 0.042 for ryr1; *p = 0.032 for atp2a1). A statistically significant difference was found among AGED mice groups by one-way ANOVA for *atp2a1* (F = 22.24, *p* < 0.0001), *atp2a2* (F = 9.979, *p* = 0.0007). Dunnett’s *post hoc* test is as follows: °vs. AGED + vehicle (0.0002 < *p*> 0.004). Data are expressed as fold-difference compared with the ADULT group, and values are expressed as mean ± SEM from a number of 4/5 mice in each group.

To gain insight into the molecular mechanism underlying the capability of BCAAs of interfering with SOCE, a gene expression analysis about the key components of SOCE has been performed on gastrocnemius (GC) muscles. As shown in Figure 4, the expression values of *ryr1* (encoding for the ryanodine receptor 1, RyR1) and *atp2a1* (encoding for the sarcoplasmic/endoplasmic reticulum Ca^2+^-transporting 1, SERCA1) genes were significantly decreased in untreated aged muscle compared with adult ones. Similarly, although not in a significant manner, *atp2a2* (encoding for the sarcoplasmic/endoplasmic reticulum Ca^2+^-Transporting 2, SERCA2) mRNA levels had reduced expression. In contrast, no alteration of *stim1/orai1/trpc1* and *cacna1s* (encoding for dihydropyridine receptor) mRNA levels were observed, suggesting that the age-related decrease in SOCE activity is not likely due to changes in the relative abundance of these SOCE components.

Interestingly, BCAA and BCAAs + 2ALA treatments efficaciously counteract the decrease of *ryr1*, *atp2a1*, and *atp2a2* gene expression, resulting in the expression values of treated animals overlapping or being even greater than the related values of adult animals. Furthermore, the same mixes induced an increase of *cacna1s* gene expression. By contrast, no effect has been observed for BCAAs + Di-ALA treatment.

**FIGURE 5:**
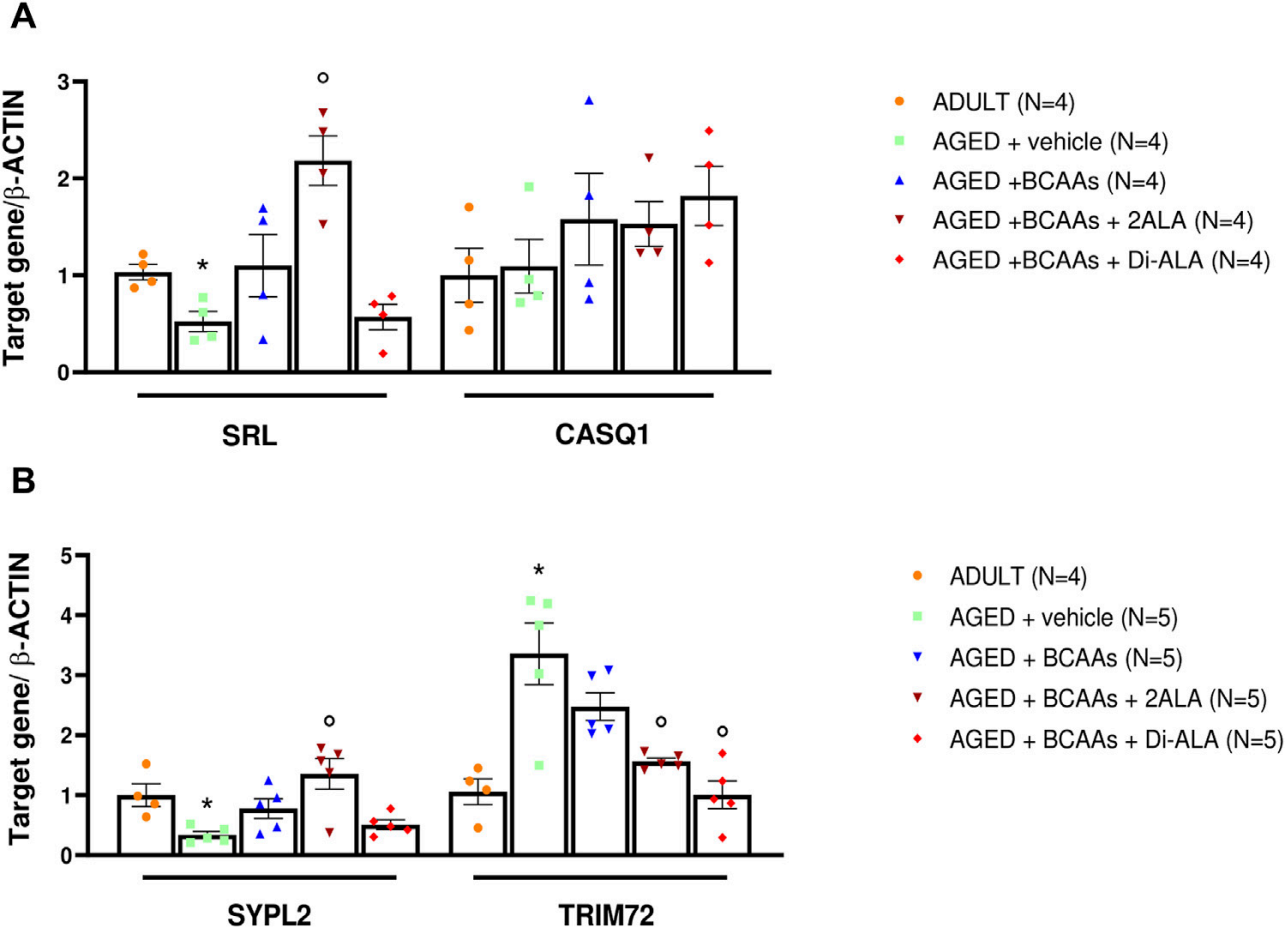
mRNA expression levels of genes encoding for calcium buffers, MG29 and MG53. Relative content of mRNA levels for **(A)** sarcalumenin (*srl*), calsequestrin (*casq1*), and **(B)** mitsugumin29 (*sypl2*) and mitsugumin 53 (*trim72)* genes normalized to *β-actin* gene in gastrocnemius (GC) muscles isolated from ADULT, AGED + vehicle, AGED + branched-chain amino acids (AGED + BCAAs), AGED + branched-chain amino acids +2 L-alanine (AGED + BCAAs + 2ALA), and AGED + branched-chain amino acids + L-alanyl-L-alanine (AGED + BCAAs + Di-ALA) groups. **(A)** For the *srl* gene, a statistically significant difference was found by unpaired Student’s t-test for AGED + vehicle vs. ADULT (*p = 0.0081). A statistically significant difference was found among AGED mice groups by one-way ANOVA (F = 12.14, *p* = 0.006). Dunnett’s *post hoc* test is as follows: °vs. AGED + vehicle (*p* = 0.0005). **(B)** For *sypl2* and *trim72* genes, a statistically significant difference was found by unpaired Student’s t-test for AGED + vehicle vs. ADULT (*p = 0.007). A statistically significant difference was found among AGED + vehicle mice groups by one-way ANOVA for *sypl2* (F = 7.867, *p* = 0.0019), *trim72* (F = 11.47, *p* = 0.0003). Dunnett’s *post hoc* test is as follows: ° vs. AGED + vehicle (0.002 < *p*< 0.02). Data are expressed as fold-difference compared with the ADULT group, and values are expressed as mean ± SEM from a number of 4/5 mice in each group.

To test whether the increased resting intracellular calcium concentration in aged skeletal muscle was associated with altered expression of the principal Ca^2+^ buffers of SR, we also evaluated expression levels of *srl* and *casq1* genes encoding for sarcalumenin and calsequestrin, respectively. In line with previous studies (O'Connell et al., 2008), there was no alteration in *casq1* expression levels, whereas a significant decrease in *srl* mRNA levels was found in the AGED + vehicle animal group compared with adult animals. The BCAAs + 2ALA mixture allowed a significant recovery of *srl* mRNA levels expression (2-fold increase vs. the expression value of AGED muscle). In contrast, no effect was exerted by BCAAs and BCAAs + Di-ALA mixture (Figure 5A). All three treatments caused a trend in increase of *casq1* mRNA levels in AGED muscles.

### 3.3 Expression analysis of MG29, MG53, and related TRP proteins

**FIGURE 6:**
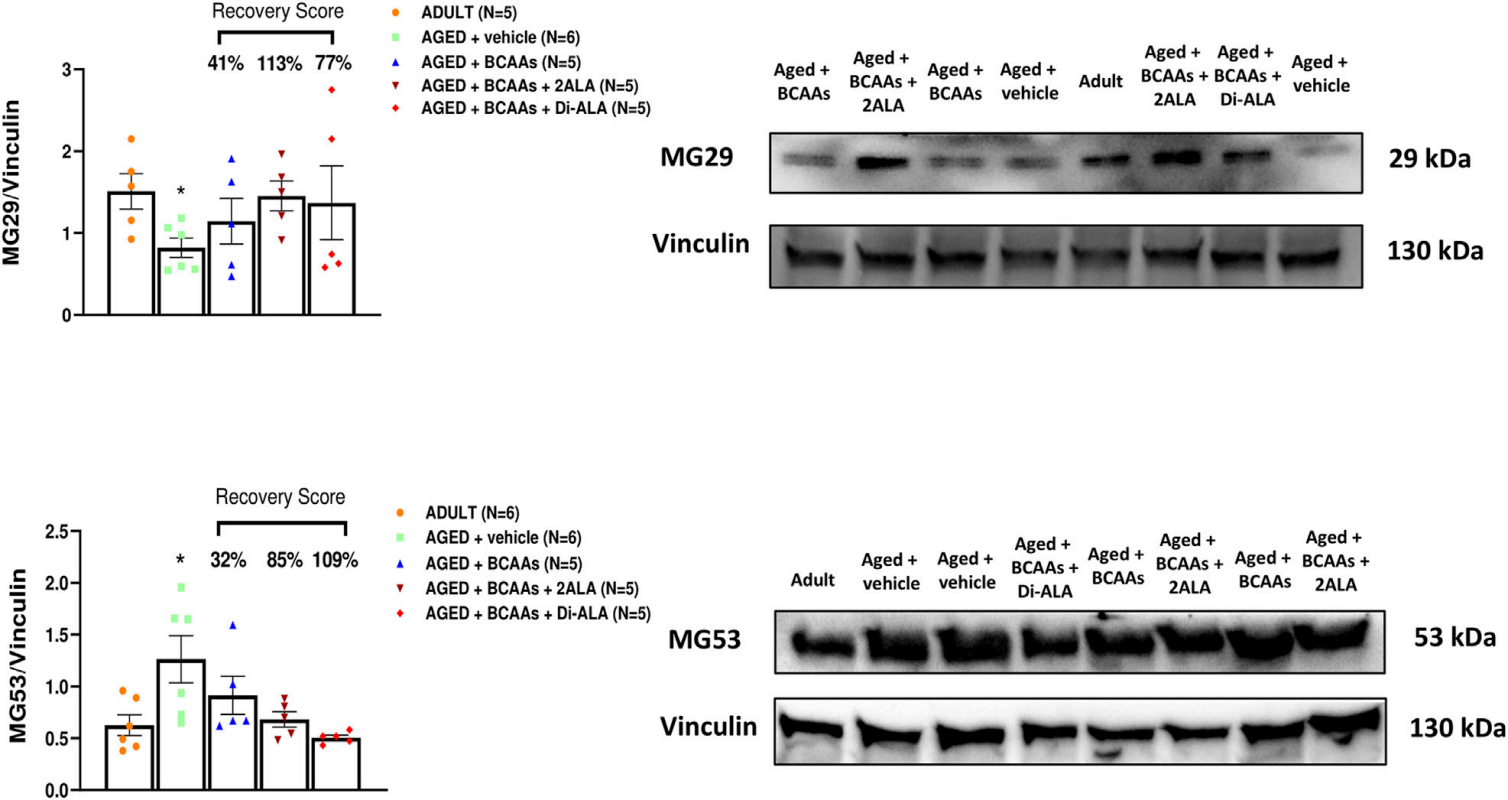
MG29 and MG53 protein expression analyses in gastrocnemius muscles. Densitometric analysis and representative Western blot of mitsugumin 29 (MG29) and mitsugumin 53 (MG53) bands normalized to vinculin of ADULT, AGED + vehicle, AGED + branched-chain amino acids (AGED + BCAAs), AGED + branched-chain amino acids +2 L-alanine (AGED + BCAAs + 2ALA), and AGED + branched-chain amino acids + L-alanyl-L-alanine (AGED + BCAAs + Di-ALA) groups. Blots were loaded by an operator unaware of the experimental groupings of samples. A statistically significant difference was found by unpaired Student’s t-test for AGED + vehicle vs. ADULT (*p = 0.0163 for MG29; *p = 0.0284 for MG53). Values are expressed as mean ± SEM from a number of 4 mice in each group. The recovery score toward the ADULT value, calculated for each treated group, is indicated above the bars.

**FIGURE 7:**
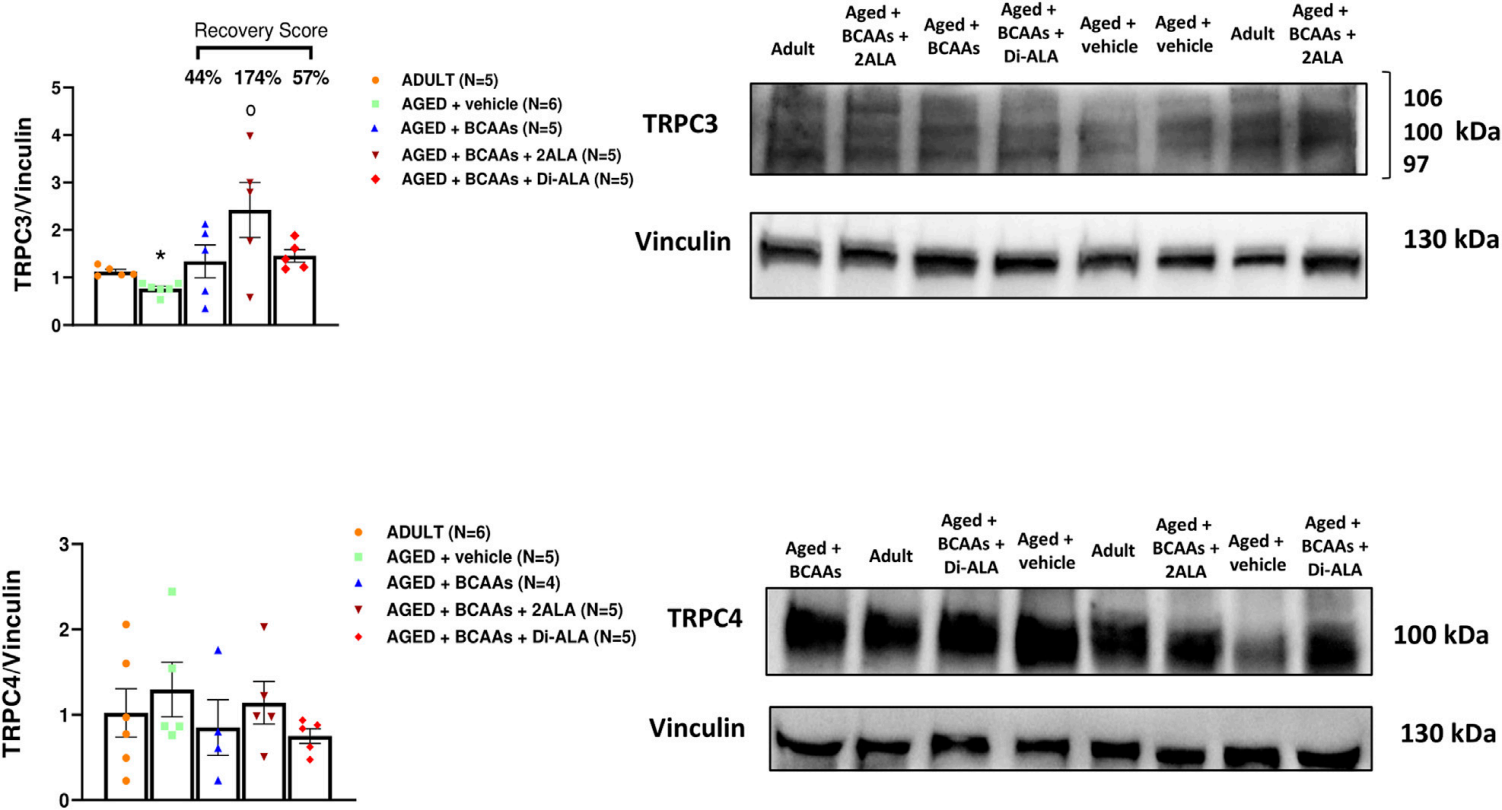
TRPC3 and TRPC4 protein expression analyses in gastrocnemius muscles. Densitometric analysis and representative Western blot of transient receptor potential cation channel subfamily C member 3 (TRPC3) and transient receptor potential cation channel subfamily C member 4 (TRPC4) bands normalized to vinculin of ADULT, AGED + vehicle, AGED + branched-chain amino acids (AGED + BCAAs), AGED + branched-chain amino acids +2 L-alanine (AGED + BCAAs + 2ALA), and AGED + branched-chain amino acids + L-alanyl-L-alanine (AGED + BCAAs + Di-ALA) groups. Blots were loaded by an operator unaware of the experimental groupings of samples. For TRPC3 protein, a statistically significant difference was found by unpaired Student’s t-test for AGED + vehicle vs. ADULT (*p = 0.0007). A statistically significant difference was found among AGED + vehicle mice groups by one-way ANOVA (F = 4.507, *p* = 0.0168). Dunnett’s *post hoc* test is as follows: °vs. AGED + vehicle (*p* = 0.0055). Values are expressed as mean ± SEM from a number of 5/6 mice in each group. The recovery score toward the ADULT value, calculated for each treated group, is indicated above the bars.

In addition to STIM1/Orai1/TRPC1/RyR1, other molecular components are recognized as players in controlling the SOCE mechanism in skeletal muscle (Gruszczynska-Biegala et al., 2021). Here, we focused on gene and protein expression of MG29 (encoded by *sypl2* gene), a synaptophysin-like protein localized at the t-tubules that acts as an SOCE and RyR1 modulator (Weisleder et al., 2006; Zhao et al., 2008; Woo et al., 2015), and MG53 (encoded by *trim72* gene) that is involved in muscle Ca^2+^ movements (Ryan et al., 2008; Cai et al., 2009; Baumann et al., 2016; Benissan-Messan et al., 2020). Furthermore, considering that MG29 is a TRPC3-interacting protein and that TRPC4 participates in SOCE (Woo et al., 2015; Antigny et al., 2017), we also assessed the expression of TRPC3 and TRPC4 at the protein levels. As shown in Figures 5B, 6, 7, we found a significant reduction of MG29 paralleled with an increase of MG53 in GC muscles of AGED + vehicle vs. ADULT mice. Although to a different extent, all formulations, particularly ALA-enriched ones, were able to counteract the alterations of MG29 and MG53 both at gene and protein expression levels. Furthermore, as shown in Figure 7, no change in TRPC4 expression was found, whereas, in parallel with MG29 reduction, we found a significant TRPC3 protein reduction in AGED mice compared to ADULT, and among treatments, only BCAAs + 2ALA treatment fully restored this reduction.

## 4 Discussion

By using an animal model of sarcopenia, this study added new pieces of evidence about the beneficial effects on dietary formulations of BCAAs on aged muscle function and unveiled a new pathway to counteract sarcopenia, that is, skeletal muscle Ca^2+^ handling mainly linked to the SOCE mechanism. The frail condition of aged mice pushed us to use a little invasive administration route for ethical reasons, that is, drinking water, in agreement with previous experiences (Capogrosso et al., 2018; Mele et al., 2019; Mantuano et al., 2020; Mantuano et al., 2021b; Mantuano et al., 2023). Although a certain interindividual variability between mice cannot be excluded *a priori*, attention has been given to verify water consumption, so dose should be adjusted in relation to body weight on a constant basis; the following observation that all the endpoint measured followed a normal distribution strengthens the view of a rather homogenous exposure in all mice.

Several studies have shown that aging processes and sarcopenia are related and/or caused by a skeletal muscle dysfunction of Ca^2+^ homeostasis (Fraysse et al., 2006; Weisleder et al., 2006; Zhao et al., 2008; Michelucci et al., 2020); however, although some hypotheses have been proposed, the mechanisms underlying these alterations are not well understood. During aging, an increase in intracellular Ca^2+^ concentration and a reduction in SOCE activity were shown to contribute to altered muscle function (Zhao et al., 2008; Brotto, 2011; Thornton et al., 2011); however, other controversial studies showed that SOCE was not compromised in muscle fibers from aged mice despite a 40% reduction in STIM1 mRNA levels (Edwards et al., 2011), and the resting [Ca^2+^]i levels were similar between young and aged fibers (Weisleder et al., 2006). In this study, we characterized the effects of sarcopenia on skeletal muscle Ca^2+^ homeostasis investigating not only the directly involved proteins but also other factors that may be of physiological relevance in the SOCE mechanism. In line with previous studies (Fraysse et al., 2006; Zhao et al., 2008; Thornton et al., 2011), our findings showed that in comparison with adult muscle, aged muscle is characterized by a two-fold increased resting [Ca^2+^]i and a reduced SOCE, thus confirming an altered Ca^2+^ homeostasis condition associated to sarcopenia, which in turn could lead to the observed alteration in muscle contractility (Batsis et al., 2014). Although no change in the expression of the main molecular components of the SOCE mechanism, such as STIM1/Orai1/TRPC1/TRPC4, was observed, aged muscle showed reduced mRNA levels of Ryr1 and SERCA1, which are the two major SR proteins controlling Ca^2+^ fluxes in skeletal muscle, and, as previously shown (O'Connell et al., 2008), of sarcalumenin, which is a luminal Ca^2+^-binding protein of the longitudinal SR that co-localizes with the SERCA Ca^2+^ pump acting as a chaperone for this protein (O'Connell et al., 2008). Altogether, these findings suggest that the expression reduction of both sarcalumenin and SERCA could contribute to age-related intracellular Ca^2+^ increase because of a less efficient mechanism of reuptake of Ca^2+^ back into the SR and of sarcalumenin-dependent Ca^2+^ buffering. In parallel, the significant reduction of RyR1 expression in aged myofibers could lead to a reduced Ca^2+^ release from the SR and therefore to a reduced signal for SOCE activation. This latter result is in line with a recent study in which authors demonstrate a decline in RyR1 content and an increased amount of degraded Ryr1 in aged muscle (Fodor et al., 2020).

Although we did not detect significant differences in Orai1 and Stim1 expression levels between adult and aged animals, we cannot rule out that concomitant aging-related mechanisms can impact their function. For instance, aged skeletal muscle is characterized by the presence of tubular aggregates (TAs) (Chevessier et al., 2005; Boncompagni et al., 2012) and unusual accumulation of SR tubes that are found in different disorders, including tubular aggregate myopathy (TAM) (Böhm et al., 2014; 2017). Recently, it has been demonstrated that Stim1 and Orai1 are accumulated in TAs in muscles of aged mice (Boncompagni et al., 2021). An attractive hypothesis is that in aged muscles, these proteins might be trapped in the SR tubes of TAs without being able to reach the proper destination in plasma membrane, thus leading to a dysfunctional SOCE.

The reduction in SOCE activity could be strictly related to aged muscle dysfunction, and thus, gaining insight into such mechanisms could reveal new targets with high therapeutic potential. Importantly, a series of studies have recently disclosed that other molecular components may contribute to the physiological SOCE mechanism in skeletal muscle in health and disease (Gruszczynska-Biegala et al., 2021). At this regard, appealing roles have been postulated for proteins MG29 and MG53. MG29 is a muscle synaptophysin-related protein, located in t-tubules and triad junction, also related to the fatigue and aging process of skeletal muscle (Takeshima et al., 1998). Indeed, MG29-deficient mice exhibit phenotypic changes in skeletal muscle like those observed in aged animals, including impaired SOCE as well as low twitch force (Pan et al., 2002; Brotto et al., 2004; Manring et al., 2014), and in aged mouse skeletal muscle, MG29 expression appears decreased (Zhao et al., 2008; O'Connell et al., 2008). Furthermore, it has been proven that MG29 is able to directly bind TRPC3, favoring its interaction with RyR1 and thus playing a role in regulating Ca^2+^ transient in skeletal muscle (Woo et al., 2015). On the other hand, the role of MG29 as a possible regulator of the SOCE mechanism in skeletal muscle was not confirmed by another study performed on adult tissue (Kurebayashi et al., 2003). In the present study, we showed that aged muscles were characterized by a reduced expression of MG29 and TRPC3 at gene and protein levels, which are features which could negatively interfere with muscle maintenance and function.

MG53 is a muscle-specific tripartite motif family protein principally expressed in cardiac and skeletal muscle and present in circulation with a key role in repair and regeneration of vital organs (Cai et al., 2009; Hu and Xiao, 2018; Whitson et al., 2021). In addition, MG53 is also a molecular component involved in cellular processes regulating excitation–contraction coupling and calcium homeostasis (Ahn et al., 2016). The MG53 protein, in fact, has been shown to interact with Orai1, favoring the SOCE mechanism, to reduce the activities of RyR1 and SERCA and to increase the expressions of TRPC3 and TRPC4 at the transcriptional level (Lee et al., 2012; Ahn et al., 2016; Benissan-Messan et al., 2020). Our findings showed an increased MG53 protein expression in aged skeletal muscle, which in relation to the observed age-related SOCE and calcium homeostasis muscle dysfunction, could be considered as an adaptative and/or compensatory response to the observed alterations. Taking into consideration the pleiotropic effects mediated by MG53, it is also possible to speculate that MG53 may also have a beneficial or protective effect on other organs affected by aging, such as the heart. Further studies are needed to confirm this hypothesis.

BCAA mixtures have been successfully used in many disease conditions characterized by a catabolic state, including muscle sarcopenia (D’Antona et al., 2010). Previous studies indicate several possible mechanisms underlying BCAA-mediated muscle protection, such as the recovery of the altered Akt/mTOR signaling (Flati et al., 2010), the improvement of mitochondrial dysfunction, or the prevention of oxidative stress characterizing sarcopenic muscles (D'Antona et al., 2010). In this setting, we previously demonstrated that BCAA supplementation improved physical performance and muscle strength, in a mouse model of physiological exercise and of disuse-induced atrophy (Mantuano et al., 2020; Mantuano et al., 2021a; Mantuano et al., 2023), and ameliorated muscle atrophy and contractile activity in aged mice (Mantuano et al., 2023). In addition, the co-administration of L-ALA, which is the main amino acid derived from the catabolism of BCAAs, resulted in the ability to enhance the availability of BCAAs by improving their ergogenic effect (Mantuano et al., 2020; Mantuano et al., 2021b).

Here, we demonstrated that after 12 weeks of supplementation, all amino acid formulations, either containing BCAAs alone or BCAAs + 2ALA or Di-ALA, were able to counteract the age-related increase of intracellular Ca^2+^ levels. Our data highlighted that all BCAA formulations also had a positive impact on muscle contractility, as shown by their ability to counteract the age-associated shift in Hz50. The significant decrease in Hz50 observed in aged mice indicates that the threshold for contraction is reached at lower stimulation frequencies, and this could be related, as in this case, to increased cytosolic calcium levels. Therefore, the amelioration of Hz50 induced by our formulations also represents an indirect index of restored calcium homeostasis. Accordingly, this was accompanied by an improvement in tetanic force, as reported in the study by Mantuano et al. (2023).

In regard of the SOCE mechanism, only the BCAAs + 2ALA formulation was able to induce a recovery of the SOCE reduction characterizing aged muscles. The protection observed at the functional level was also detectable on the expression profile of proteins involved in the SOCE mechanism and calcium buffering, such as MG29, MG53, TRPC3, and sarcalumenin proteins. In all cases, particularly at the transcript level, the BCAAs + 2ALA formulation, and to a lesser extent, even BCAAs alone, were effective in restoring the alteration age-related muscle features under investigation. Our findings further support the hypothesis that BCAA formulations have a protective potential to counteract aging-related skeletal muscle functional alterations, revealing new actions of BCAAs interfering with Ca^2+^ keys pathways. Based on our findings, we might speculate that the reduction of resting Ca^2+^ concentration together with the partial or total recovery of the SOCE mechanism, in terms of activity and expression of the various protein components, might be at the basis of the improved muscle performance induced by BCAA formulations. As the increase of MG53 expression observed during aging is considered as a compensatory mechanism to protect the membrane from oxidative stress (Ryan et al., 2008; Hord et al., 2016; Wang et al., 2021), the trend of decrease of MG53 expression induced by BCAAs could provide further evidence of the capability of these amino acids to protect skeletal muscle.

Finally, the known BCAA capability of activating mTOR and improving proteostasis (Maki et al., 2012) could play a role in mediating the observed beneficial effects. Indeed, it has been recently demonstrated that a long-term exercise improves the impaired proteostatic mechanism *via* modulation of mTOR (Fuqua et al., 2023) by enhancing its sensitivity to amino acids, particularly leucine (D’Hulst et al., 2022). Thus, we might hypothesize that BCAA formulations, also containing leucine, can in part resemble the effect of exercise, preventing or limiting the improper accumulation of Stim1 and Orai1, for example, in TAs and ultimately ameliorating the SOCE mechanism.

In conclusion, skeletal muscle is among the most age-sensitive tissues in mammal organisms. In this context, the results we obtained in this study corroborate the hypothesis that Ca^2+^ homeostasis and SOCE dysfunction may contribute to muscle weakness during aging. In addition, our results support the potential usefulness of BCAAs in combination with L-alanine. In particular, the formulation with the greatest efficacy on the examined parameters was BCAAs + 2ALA, containing BCAAs (2:1:1) + 2 L-ALA, so its use may be an effective dietary strategy to contrast age-related muscle alterations and sarcopenia. Based on our results, 2ALA or Di-ALA co-administration could be hypothesized to have a fiber-type specific action on fast-twitch muscles (i.e., EDL) and on muscles with similar composition (i.e., GC) in aged mice. Interestingly, in fast glycolytic fibers, the enhancement of BCAA effectiveness provided by ALA may be related to the increased availability of this amino acid to participate into the glucose/alanine cycle, which is a key interplay for glycolytic energy metabolism between the liver and skeletal muscle, and to serve as an energy source (Sarabhai and Roden, 2019). However, although calcium-related functional indices (i.e., Hz50) were not altered in the slow-twitch SOL muscle, a potential limitation of our study is represented by the low amount of tissue that did not allow us to perform comparative molecular analyses on this muscle to ascertain fiber type-dependent effects of BCAA formulations on genes involved in calcium homeostasis. In addition, the present results do not allow us to rule out possible additional effects of BCAAs on other players that may contribute to calcium dyshomeostasis in our model, for example, the Ca^2+^ content in SR and/or in mitochondria, which are key aspects that can be addressed in future dedicated experiments.

Overall, this new nutritional strategy could produce a better quality of life and resistance to frailty in elderly patients and in other muscle-wasting-related conditions.

## Figures and Tables

**Table:** 

**BCAAs**	Branched-chain amino acids
**CASQ1**	Calsequestrin 1
**DHPR**	Dihydropyridine receptor
**Di-ALA**	L-alanyl-L-alanine
**EAAs**	Essential amino acids
**EDL**	Extensor digitorum longus muscle
**FDB**	Flexor digitorum brevis muscle
**GC**	Gastrocnemius muscle
**L-ALA**	L-alanine
**MG29**	Mitsugumin29
**MG53**	Mitsugumin53
**mTORC1**	Mammalian target of rapamycin complex 1
**ORAI1**	Calcium release-activated calcium modulator 1
**PVDF**	Polyvinylidene difluoride
**Ryr1**	Ryanodine receptor type 1
**SERCA**	Sarcoplasmic/endoplasmic reticulum Ca^2+^ transporter
**SOCE**	Store-operated calcium entry
**SR**	Sarcoplasmic reticulum
**SRL**	Sarcalumenin
**STIM1**	Stromal interaction molecule 1
**TRPC1**	Transient receptor potential cation channel, subfamily C, member 1
**TRPC3**	Transient receptor potential cation channel, subfamily C, member 3
**TRPC4**	Transient receptor potential cation channel, subfamily C, member 4

## Data Availability

The original contributions presented in the study are included in the article, further inquiries can be directed to the corresponding author.
